# Trans-β-galactosidase activity of pig enzymes embedded in the small intestinal brush border membrane vesicles

**DOI:** 10.1038/s41598-018-37582-8

**Published:** 2019-01-30

**Authors:** Lesbia Cristina Julio-Gonzalez, Oswaldo Hernandez-Hernandez, F. Javier Moreno, Agustín Olano, Maria Luisa Jimeno, Nieves Corzo

**Affiliations:** 1 Instituto de Investigación en Ciencias de la Alimentación, CIAL (CSIC-UAM), CEI (UAM+CSIC), Nicolás Cabrera 9, 28049 Madrid, Spain; 2 Centro de Química Orgánica “Lora Tamayo” (CSIC), Juan de la Cierva 3, 28006 Madrid, Spain

## Abstract

This work highlights the utility of brush border membrane vesicles (BBMV) from the pig small intestine as a reliable model for gathering information about the reaction mechanisms involved in the human digestion of dietary carbohydrates. Concretely, the elucidation of the transgalactosylation mechanism of pig BBMV to synthesize prebiotic galacto-oligosaccharides (GOS) is provided, unravelling the catalytic activity of mammalian small intestinal β-galactosidase towards the hydrolysis of GOS. This study reveals that pig BBMV preferably synthesizes GOS linked by β-(1 → 3) bonds, since major tri- and disaccharide were produced by the transfer of a galactose unit to the C-3 of the non-reducing moiety of lactose and to the C-3 of glucose, respectively. Therefore, these results point out that dietary GOS having β-(1 → 3) as predominant glycosidic linkages could be more prone to hydrolysis by mammalian intestinal digestive enzymes as compared to those linked by β-(1 → 2), β-(1 → 4), β-(1 ↔ 1) or β-(1 → 6). Given that these data are the first evidence on the transglycosylation activity of mammalian small intestinal glycosidases, findings contained in this work could be crucial for future studies investigating the structure-small intestinal digestibility relationship of a great variety of available prebiotics, as well as for designing tailored fully non-digestible GOS.

## Introduction

Since prebiotics were first defined as “non-digestible food ingredients that beneficially affect the host by selectively stimulating the growth and/or activity of one or a limited number of bacteria in the colon, thus improving host health”^1^, a considerable number of carbohydrates varying in monosaccharides composition and order, configuration and position of glycosidic linkages have been proposed as potential prebiotics. However, available prebiotic carbohydrates with proven clinical efficacy are only inulin, fructooligosaccharides (FOS), galactooligosaccharides (GOS) and lactulose^2^. In particular, GOS have attracted increasing interest from academics and industry researchers mainly because of the presence of galactose-based oligosaccharides in human milk and the relative structural similarity between commercial GOS and human milk oligosaccharides (HMOs)^3^.

Commercial dietary GOS are usually synthetized by enzymatic transglycosylation from lactose catalyzed by β-galactosidases from bacteria, fungi or yeasts and comprised of a complex mixture of oligosaccharides that can vary from 1 to 8 galactose units and a terminal glucose^4^. Recently, a considerable number of commercial GOS have been comprehensively characterized, all showing the same set of β-D-Gal*p*-(1 → x)-D-Glc*p* (x = 2; 3; 4; and 6) disaccharides and β-D-Gal*p*-(1 → x)-[β-D-Gal*p*-(1 → y)-]D-Glc*p* (x, y = 2, 4; 2, 6; and 3, 6) branched trisaccharides, although in rather different molar ratios^5^. The variation in composition of commercial GOS samples is mainly due to the β-galactosidase origin^5,6^.

A common notion regarding prebiotics is that they are resistant to the body’s enzymes, are not digested as they travel through the digestive system, and reach the colon unaltered. However, there are some *in vivo* studies performed with neonatal and growing rats showing that GOS are selectively digested in the small intestine^7,8^, challenging the assumption that GOS reach the colon with their original structure fully intact. Similar findings have been recently reported with *in vitro* digestion models using a rat small intestinal extract^9,10^.

The small intestinal brush border enzymes in mammals include a large number of hydrolases, notably peptidases and glycosidases, that are expressed by the enterocyte to maintain a high digestive capacity of its apical brush border in the environment of the intestinal lumen^11,12^. Although intestinal disaccharidase activities, especially β-galactosidase, gradually decrease during aging of mammals, the high physiological and anatomical similarity of the pig and human digestive tracts^13,14^ makes the use of brush border membrane vesicles (BBMV) of the pig small intestine an ideal model for gathering information about the reaction mechanisms involved in the human digestion of GOS as it has been previously demonstrated for the digestion of HMOs^15^. Considering that enzymes are, basically, catalysts and, under appropriate experimental conditions, can catalyze reversible reactions in either direction, there is a reasonable probability that the most abundant glycosidic linkages, formed when mammalian intestinal β-galactosidase act as transgalactosidase, will be preferentially broken under hydrolytic conditions. In fact, studies on microbial β-galactosidases have shown that hydrolysis and transglycosylation occur simultaneously at the same active site of the enzyme. Then, the reaction equilibrium can easily be shifted to favor transglycosylation by decreasing water activity in the reaction mixture, which is mainly achieved by incubating the enzyme with highly concentrated lactose solution^16^.

Among the numerous studies reported on transgalactosidase activities of β-galactosidases from different sources^17,18^, no data are available on GOS synthesized by mammalian intestinal β-galactosidase despite the potential biological relevance to understand GOS digestion fate. This work aims to fill this gap by elucidating the mechanisms of transgalactosylation activity of pig enzymes embedded in the small intestinal BBMV by using supraphysiological concentrations of lactose. The structure-function information obtained in this study may lead to a predictive understanding about specific GOS structures that can resist the small intestinal digestion.

## Results

### Ability of pig small intestinal brush border membrane vesicles (BBMV) to synthesize GOS

**Figure 1:**
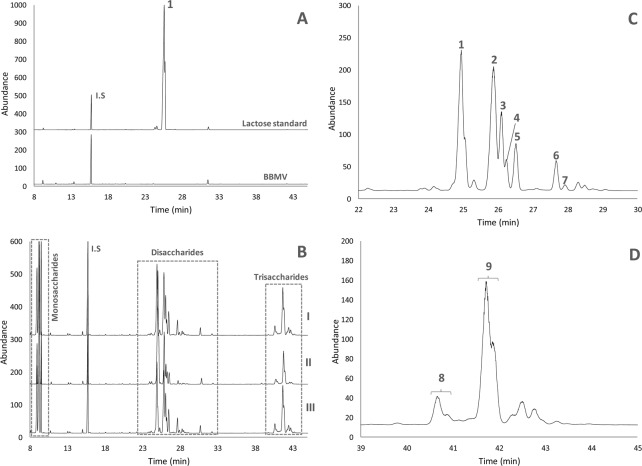
Profiles obtained by gas chromatography with a flame ionization detector (GC-FID) of TMSO derivatives of: (**A**) lactose standard before incubation with BBMV and the control BBMV alone (not incubated with lactose), (**B**) GOS synthesized by three isolated individual pig small BBMV (I, II and III), (**C**) GOS disaccharide and (**D**) GOS trisaccharide fractions synthesized by an isolated individual pig small intestinal BBMV (III). Labelled peaks are described in the text.

The three isolated individual pig small intestinal BBMV showed a considerable transgalactosylation activity under the experimental conditions used in this study, allowing the efficient synthesis of GOS with degree of polymerization (DP) 2 and 3 (Fig. 1B–D). The respective GOS profiles obtained by GC-FID did not show any qualitative difference (Fig. 1B), indicating an identical transgalactosylation mechanism for each individual intestinal BBMV. Concerning disaccharide fraction, in addition to lactose (peak labelled as 1 in Fig. 1C), another six peaks could be identified (peaks from 2 to 7, Fig. 1C), whereas a lower variability was found in the trisaccharide fraction with only two compounds of significant dissimilar abundance clearly detected (peaks 8 and 9, Fig. 1D). Any potential interference in the GC-FID analysis coming from BBMV material, which was removed before the derivatization step, was ruled out according to the blank sample chromatograms shown in Fig. 1A.

Table 1 shows the quantitative data of monosaccharides, lactose and synthesized GOS by the three individual small intestinal BBMV throughout the enzymatic reaction. Overall, the highest content of GOS and the lowest level of lactose were obtained by using pig small intestinal BBMV termed as 3, followed by pig BBMV 2 and pig BBMV 1, respectively. The capacity to hydrolyze lactose and, subsequently, to synthesize GOS exhibited by the three individual BBMV was consistent with their corresponding β-galactosidase activity values described in Methods section. These quantitative differences were more remarkable at the beginning of the enzymatic reactions, whereas the levels of total GOS synthesized by the individual BBMV were more similar at the end of the reaction (6 hours). Lastly, the content in GOS disaccharides (not including lactose) and trisaccharides was balanced in all cases.

**Table 1:** Concentration (mg/100 mg of initial lactose) of monosaccharides, lactose and synthesized GOS upon transgalactosylation reaction catalyzed by three isolated individual pig small intestinal BBMV (I, II and III).

Small intestinal BBMV	Incubation time (h)	Monosaccharides (mg/100 mg lactose)		Lactose (mg/100 mg lactose)	GOS (mg/100 mg lactose)		Total GOS
		Galactose	Glucose		Disaccharides	Trisaccharides	
Pig I	0	0.0 (0.0^a^)	0.0 (0.0)	100 (0.0)	0.0 (0.0)	0.0 (0.0)	0.0
	2	2.2 (0.3)	5.1 (1.0)	63.0 (8.8)	6.1 (1.4)	6.2 (0.9)	12.3
	4	3.8 (0.2)	12.1 (0.2)	48.0 (1.2)	14.3 (0.2)	11.6 (0.5)	25.9
	6	5.0 (0.4)	15.4 (0.5)	33.7 (3.1)	17.6 (0.4)	14.5 (0.1)	32.1
Pig II	0	0.0 (0.0)	0.0 (0.0)	100 (0.0)	0.0 (0.0)	0.0 (0.0)	0.0
	2	2.0 (0.1)	6.7 (0.2)	63.6 (4.9)	8.4 (0.1)	6.4 (0.1)	14.8
	4	4.2 (0.2)	13.3 (0.6)	52.7 (3.9)	18.9 (0.5)	15.9 (0.9)	34.8
	6	4.8 (0.1)	15.9 (0.8)	28.9 (2.8)	20.4 (1.6)	17.3 (1.3)	37.7
Pig III	0	0.0 (0.0)	0.0 (0.0)	100 (0.0)	0.0 (0.0)	0.0 (0.0)	0.0
	2	4.7 (0.0)	16.3 (0.5)	37.9 (3.1)	20.6 (0.6)	16.3 (0.1)	36.9
	4	6.2 (0.1)	21.9 (1.0)	23.5 (3.7)	25.4 (1.5)	20.9 (1.1)	46.3
	6	7.5 (0.0)	25.3 (0.0)	13.6 (0.0)	27.0 (0.0)	21.0 (0.0)	48.0

^a^Standard deviation values (*n* = 2).

### Structural characterization of GOS synthesized by pig small intestinal brush border membrane vesicles (BBMV)

To accomplish a comprehensive structural characterization of the main synthesized GOS, the disaccharide and trisaccharide fractions were isolated by HILIC-RID for further characterization by NMR. The purity degree of the isolated fractions, checked by GC-FID, was considered to be appropriate for their subsequent NMR analysis, since the purified fractions contained the major GOS disaccharide (labelled as peaks 2 and 4 in Fig. 1C), and trisaccharides (peaks 8 and 9 in Fig. 1D), respectively. NMR characterizations were performed by the combined use of 1D and 2D [^1^H, ^1^H] and [^1^H, ^13^C] NMR experiments (gCOSY, TOCSY, ROESY, multiplicity-edited gHSQC and gHMBC). ^1^H and ^13^C NMR chemical shifts observed are summarized in Table 2. Full set of spectra are available in the Supporting Information (Figures S1–S10).

**Table 2:** ^1^H (500 MHz), ^13^C (125 MHz) NMR chemical shifts^a^ (δ, ppm) and coupling constants (J in Hz, in parentheses) of major disaccharide and trisaccharide.

		Structure 1 (β-Gal-(1 → 3)-Glc)				Structure 2 (β-Gal-(1 → 3)-β-Gal-(1 → 4)-Glc)			
		β (0.6)		α (0.4)		β (0.6)		α (0.4)	
		δ_C_	δ_H_	δ_C_	δ_H_	δ_C_	δ_H_	δ_C_	δ_H_
Gal	1	104.3	4.65 (8.1)	104.4	4.64 (8.1)	105.2	4.61 (8.1)	105.2	4.61 (8.1)
	2	72.2	3.60	72.1	3.58	72.2	3.61	71.9	3.61
	3	73.5	3.68	73.6	3.70	73.5	3.67	73.4	3.67
	4	69.6	3.92	69.6	3.92	69.6	3.92	69.5	3.92
	5	76.3	3.70	76.3	3.70	76.3	3.69	76.0	3.69
	6	62.0	3.75, 3.80	62.0	3.75, 3.80	62.0	3.73, 3.80	61.9	3.73, 3.80
Gal	1					103.4	4.51 (8.1)	103.4	4.51 (8.1)
	2					71.1	3.72	71.1	3.72
	3					82.8	3.84	82.8	3.84
	4					69.3	4.20	69.3	4.20
	5					75.9	3.75	75.9	3.75
	6					61.9	3.73, 3.80	61.9	3.73, 3.80
Glc	1	96.6	4.68 (8.1)	93.0	5.24 (3.8)	96.7	4.67 (8.1)	92.7	5.23 (3.8)
	2	74.7	3.43	71.9	3.73	74.7	3.29	72.1	3.58
	3	85.5	3.74	83.4	3.91	75.3	3.65	72.3	3.84
	4	69.2	3.51	69.2	3.52	79.0	3.68	79.2	3.67
	5	76.5	3.50	72.2	3.89	75.7	3.61	71.0	3.95
	6	61.7	3.78, 3.92	61.5	3.80, 3.86	61.0	3.81, 3.96	60.9	3.82, 3.88

^a^Chemical shifts in ppm for ^1^H (δ_H_) and ^13^C (δ_C_) spectra were determined relative to an external standard of sodium [2, 2, 3, 3-^2^H_4_]-3-(trimethylsilyl) propanoate in D_2_O (δ_H_ 0.00 ppm) and 1,4-dioxane. (δ_C_ 67.40 ppm) in D_2_O, respectively.

### GOS-disaccharide fraction

**Figure 2:**
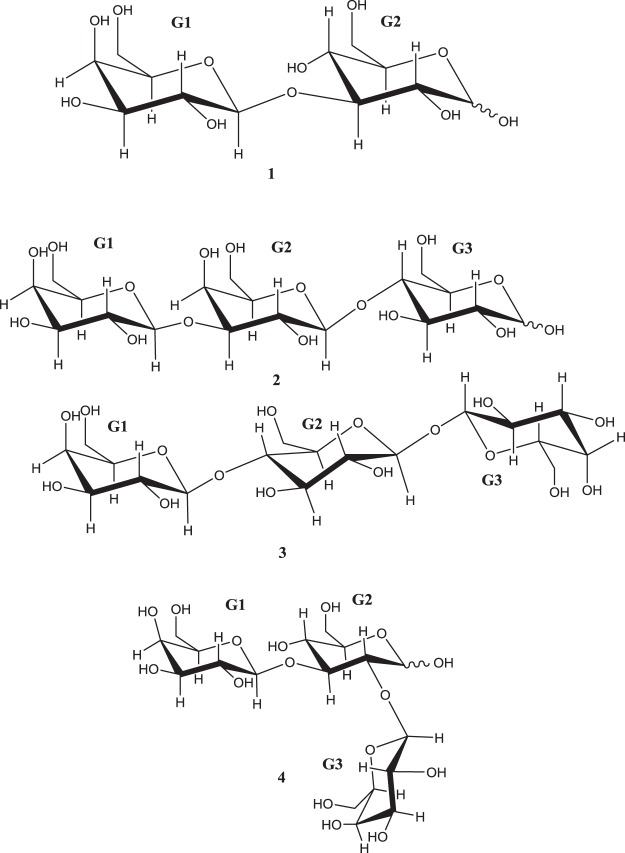
Structures of the oligosaccharides characterized by NMR from GOS-disaccharide and GOS-trisaccharide fractions. (1) β-Gal-(1 → 3)-Glc; (2) β-Gal-(1 → 3)-β-Gal-(1 → 4)-Glc; (3) β-Gal-(1 → 4)-β-Glc-(1 ↔ 1)-β-Gal; (4) β-Gal-(1 → 3)-Glc-(2 → 1)-β-Gal.

The 1D ^1^H NMR spectrum of the major GOS disaccharide (termed as 1 in Fig. 2) showed two sets of two doublets in the anomeric region (δ5.24, δ4.64 and δ4.68, δ4.65). Besides, the 1D ^13^C NMR spectrum also showed two sets of two resonances in the anomeric region (δ104.3, δ96.6 and δ104.4, δ93.0). These data are compatible with the existence of the two anomeric forms of the reducing terminal unit.

The 2D COSY and TOCSY spectra revealed the ^1^H signals of a unit of galactopyranose and a unit of glucopyranose that allowed us to correlate them with the corresponding carbon signals in the multiplicity-edited gHSQC spectra. To assign the configuration of each anomeric center, the values of the vicinal coupling constants for the anomeric protons were used. These results were consistent with the structure of a disaccharide with the G1 unit being β-D-galactopyranosyl and the reducing terminal G2 unit glucose with β and α forms in a 60:40 ratio. The position of the glycosidic linkage was analyzed as follows: for the major isomer, gHMBC showed correlations between the anomeric proton of G1 (4.65 ppm) and the C3 carbon of the terminal glucose G2 (85.5 ppm), and between the anomeric carbon of G1 (104.3 ppm) and the H3 of G2 (3.74 ppm). The minor isomer gHMBC showed correlations between the anomeric proton of G1 (4.64 ppm) and the C3 carbon of the terminal α -glucose G2 (83.4 ppm), and between the anomeric carbon of G1 (104.4 ppm) and the H3 of G2 (3.91 ppm). These data are compatible with a beta 1–3 linkage. Finally, Structure **1** was established as β-D-Gal-(1 → 3)-D-Glc. The complete ^1^H and ^13^C sets of chemical shifts (Table 2) are identical to those published in the literature^19^.

To complete the characterization of the disaccharide fraction, a tentative identification of the less abundant disaccharides was performed by GC-MS (Figure S11) with the exception of lactose that was identified by comparison with its commercial standard. Remarkably, all identified disaccharides were β-galactosyl-glucoses linked by (1 → 2), (1 → 3), (1 → 4) (lactose) or (1 → 6) (allolactose) bonds, whilst no galactobioses (galactosyl-galactoses) were found. The tentative identification of β-galactosyl-glucoses was confirmed, in a first step, by the combined presence of the ions at *m/z* 204, which is indicative of disaccharides with glycosidic linkages in β configuration, and at *m/z* 217, indicative of the presence of a glucose unit at the reducing end. In the case of β-Gal-(1 → 3)-Glc (peaks 2 and 4 in Fig. 1C), although previously identified by NMR (Fig. 2 and Table 2), its mass spectra were characterized by the high ratio of ions at *m/z* 205/204 and the high abundance of the ion at *m/z* 244, which are typical for disaccharides with (1 → 3) linkages. Mass spectra of GC peaks 3 and 5 showed a high intensity of the ion at *m/z* 319, which can be originated by the loss of TMSOH group from the ion at *m/z* 409 corresponding to C-3, 4, 5 and 6 of the reductive end. Consequently, the ratio of ions at *m/z* 319/361 was higher than 1. All these patterns are characteristic for (1 → 2) linkages. Finally, mass spectra of GC peaks 6 and 7 showed a relatively high intensity of the ion at *m/z* 422, corresponding to C-1, 2, 3 and 4 of the oxime chain, and a relatively low abundance of the ion at *m/z* 205, both indicative of (1 → 6) linkages^20,21^.

### GOS-trisaccharide fraction

The 1D ^1^H NMR spectrum of the major GOS trisaccharide (termed as 2 in Fig. 2) showed two sets of three doublets in the anomeric region (δ4.61, δ4.51, δ4.67) and (δ4.61, δ4.51, δ5.23) in a 60:40 ratio. In addition, the 1D ^13^C NMR spectrum showed signals corresponding to two sets of 18 carbons, each of them including three anomeric carbons (δ105.2, δ103.4, and δ96.7 for the major anomeric form), and (δ105.2, δ103.4, and δ92.7 for the minor anomeric form). These values are indicative of the presence of a trisaccharide with three hexose sugars in the structure. A multiplicity-edited gHSQC spectrum was used to link the carbon signals to the corresponding proton resonances. 2D COSY and TOCSY experiments and the coupling constant values revealed ^1^H signals consistent with the structure of a trisaccharide with G1 and G2 units being β-D-galactopyranosyl and the reducing terminal G3 unit a glucose with β and α forms in a 60:40 ratio. The position of the glycosidic linkage was analyzed as follows: for the major isomer, gHMBC showed correlations between the anomeric proton of G1 (4.61 ppm) and the C3 carbon of G2 (82.8 ppm), between the anomeric carbon of G1 (105.2 ppm) and the H3 of G2 (3.84 ppm), between the anomeric proton of G2 (4.51 ppm) and the C4 carbon of the terminal glucose G3 (79.0 ppm), and between the anomeric carbon of G2 (103.4 ppm) and the H4 of G3 (3.68 ppm). Similar correlations for the minor isomer were found. Finally, the structure of the main trisaccharide 2 was assigned to β-D-Gal-(1 → 3)-β-D-Gal-(1 → 4)-D-Glc. The complete ^1^H and ^13^C sets of chemical shifts (Table 2) are identical to those published in the literature^19^.

**Figure 3:**
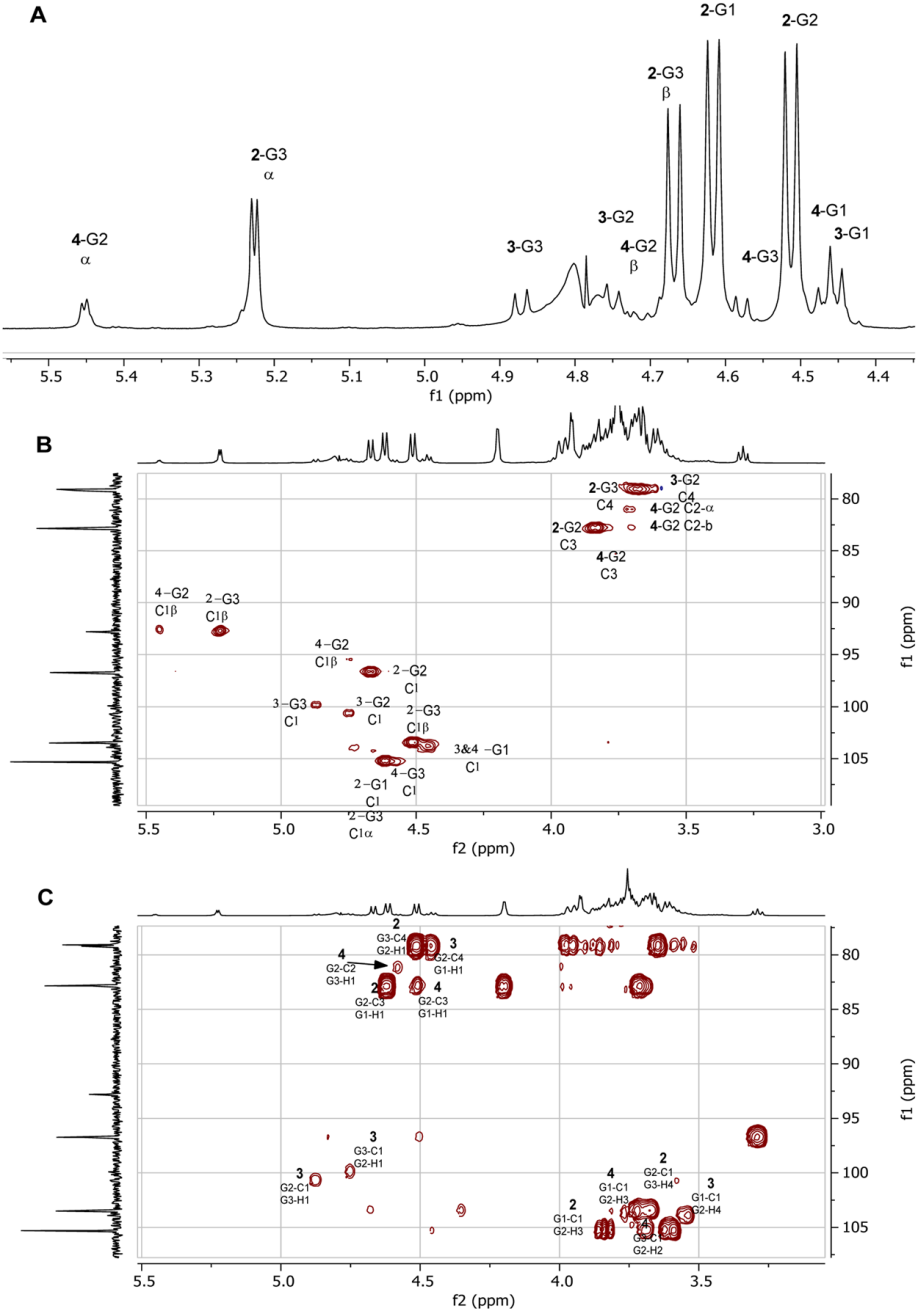
NMR spectra of GOS-trisaccharide fraction. (**A**) 1D-^1^H anomeric region of degree of polymerization (DP) 3. (**B**) gHSQC contour plot expansion showing relevant correlations. (**C**) gHSQC contour plot expansion showing inter-residue correlations.

At least two additional minor trisaccharides were detected in the 1D ^1^H NMR and 1D ^13^C NMR spectra of the purified DP3 fraction. Due to considerable overlapping of the signals, only a tentatively assignation based in anomeric protons and carbon chemical shifts and coupling constants could be carried out. A deconvolution of the anomeric region of the ^1^H spectrum allowed to identify signals corresponding to both of them (Fig. 3A).

The minor trisaccharide, termed as 3 in Fig. 2, showed three correlations bands (δ4.87, J = 8.1 Hz; δ99.8), (δ4.74, J = 7.9 Hz; δ100.6) and (δ4.47, J = 7.8 Hz; δ103.8) in the anomeric region of gHSQC spectrum (Fig. 3B). The gHMBC spectrum showed correlations between the anomeric proton of G2 (4.74 ppm) and the C1 carbon of G3 (99.8 ppm), between the anomeric carbon of G2 (100.6 ppm) and H1 of G3 (4.87 ppm), and between H1 of G1 (4.47 ppm) and C4 of G2 (78.7 ppm) (Fig. 3C). These correlations indicated a 1-1-linkage between G2 and G3 and a 1–4 linkage between the G1 and G2. 2D COSY, TOCSY and ROESY experiments and the coupling constant values revealed ^1^H signals consistent with the structure of a trisaccharide with the G1 and G3 units being β-D-galactopyranosyl and G2 being β-D-glucopyranosyl. The chemical shifts measured in the TOCSY experiment for G1 and G2 are quite similar to the model reference β-D-Gal*p*-(1 → 4)-β-D-Glcp^19^. Consequently, the structure of the trisaccharide 3 (Fig. 2) was tentatively assigned to β-D-Gal-(1 → 4)-β-D-Glc-(1 → 1)-β-D-Gal.

Another minor trisaccharide, termed as 4 in Fig. 2, showed three correlations (δ5.45, J = 3.3 Hz; δ92.6), (δ4.58, J = 7.7 Hz; δ105.3) and (δ4.48, J = 7.8 Hz; δ103.8) in the anomeric region of gHSQC spectrum (Fig. 3B). The ^1^H doublet at 5.45 ppm and the ^13^C chemical shift at 92.6 ppm indicated a 2-substituted reducing α-D- glucopyranosyl residue. The 2D COSY, TOCSY and ROESY experiments revealed ^1^H signals consistent with the structure of a trisaccharide with the G1 and G3 units being β-D-galactopyranosyl and G2 being D-glucopyranosyl. gHMBC spectrum showed correlations between the anomeric proton of G3 (4.58 ppm) and the C2 carbon of the glucose G2 (81.1 ppm), and between the C3 of G2 (82.8 ppm) and the H1 of G1 (4.48 ppm) (Fig. 3C). Some signals from the β-D-glucopyranosyl form can be observed from the TOCSY spectrum, but the anomeric proton (aprox 4.7–4.8) could not be detected due to the effect of suppression of the HOD peak. These correlations indicated a 1–3-linkage between G1 and G2 and a 1–2 linkage between G3 and G2. The measured ^1^H and ^13^C chemical shifts are quite similar to the model references β-D-Galp-(1 → 3)-β-D-Glc*p* and β-D-Gal*p*-(1 → 2)-D-Glc*p*^19^. Therefore, the structure the trisaccharide **4** was tentatively assigned to β-D-Gal-(1 → 3)-D-Glc-(2 → 1)-β-D-Gal.

The main trisaccharides found in commercial dietary GOS are typically 3′-, 4′- and/or 6′-galactosyl-lactose^5^. To investigate the potential presence of 4′- and 6′-galactosyl-lactose, the GC retention indexes of their respective commercial standards were compared to those of the GOS trisaccharide fraction synthesized by the three individual BBMV. Based on the GC behavior, the presence of 6′-galactosyl-lactose could be ruled out, whereas the 4′-galactosyl-lactose might be present but only at trace amounts as a shoulder of peak 9 (Fig. 1D).

### Quantification of structurally characterized GOS

Table 3 summarizes the quantifiable disaccharide and trisaccharide structures, as well as their individual content and percentage distribution within the same DP obtained by the synthesis catalyzed by the studied pig small intestinal BBMV after 6 hours of the transgalactosylation reaction. Regarding the GOS disaccharide fraction, apart from lactose, the main compound was β-Gal-(1 → 3)-Glc, representing between 23–36% of the total disaccharide fraction, followed by β-Gal-(1 → 2)-Glc (8–21%) and β-Gal-(1 → 6)-Glc (1–6%). The predominant trisaccharide was β-Gal-(1 → 3)-β-Gal-(1 → 4)-Glc (also named 3′-galactosyl-lactose), followed very distantly by a combination of two trisaccharides, i.e. β-Gal-(1 → 4)-β-Glc-(1 ↔ 1)-β-Gal and β-Gal-(1 → 3)-Glc-(2 → 1)-β-Gal, with a percentage distribution of 70–79% and 14%, respectively. Lastly, no trisaccharides having the β-(1 → 6) glycosidic linkage could be identified in this study (Fig. 1D).

**Table 3:** Chemical structure, content (mg/100 mg of initial lactose) and percentage distribution within the same degree of polymerization (DP) of GOS synthesized upon 6 hours of transgalactosylation reaction catalyzed by three isolated individual pig small intestinal BBMV (I, II and III).

DP	GC-FID peak number^a^	Chemical structure	Content (mg/100 mg lactose)			Percentage distribution per DP		
			Pig BBMV I	Pig BBMV II	Pig BBMV III	Pig BBMV I	Pig BBMV II	Pig BBMV III
2	1	β-Gal-(1 → 4)-Glc	33.7 (3.1^d^)	28.9 (2.8)	13.6 (0.0)	66.0 (1.3)	60.8 (0.3)	35.3 (0.3)
	2^b^	β-Gal-(1 → 3)-Glc *E*	10.0 (0.5)	11.2 (1.0)	11.8 (0.3)	19.6 (0.5)	22.0 (0.1)	31.2 (0.2)
	4^b^	β-Gal-(1 → 3)-Glc Z	1.7 (0.2)	2.0 (0.2)	1.7 (0.1)	3.4(0.1)	4.0 (0.1)	4.4 (0.1)
	3^c^	β-Gal-(1 → 2)-Glc *E*	2.4 (0.0)	3.6 (0.2)	4.8 (0.3)	4.8 (0.3)	7.1 (0.2)	12.8 (0.4)
	5^c^	β-Gal-(1 → 2)-Glc Z	1.7 (0.1)	2.1 (0.1)	3.1 (0.2)	3.3 (0.1)	4.2 (0.1)	8.1 (0.2)
	6^c^	β-Gal-(1 → 6)-Glc *E*	0.5 (0.0)	0.7 (0.0)	2.0 (0.0)	0.9 (0.2)	1.4 (0.2)	5.2 (0.1)
	7^c^	β-Gal-(1 → 6)-Glc *Z*	0.2 (0.0)	0.03 (0.0)	0.2 (0.0)	0.4 (0.1)	0.06 (0.0)	0.6 (0.0)
3	8^b^	β-Gal-(1 → 4)-β-Glc-(1 ↔ 1)-β-Gal β-Gal-(1 → 3)-Glc-(2 → 1)-β-Gal	1.7 (0.1)	2.4 (0.2)	2.6 (0.2)	14.1 (0.3)	14.2 (0.1)	14.1 (0.1)
	9^b^	β-Gal-(1 → 3)-β-Gal-(1 → 4)-Glc	9.7 (0.8)	12.7 (1.0)	13.0 (0.8)	78.5 (1.4)	73.9 (0.2)	69.8 (0.5)

^a^Labelled peaks according to Fig. 1.

^b^Identification by NMR.

^c^Tentative identification by GC-MS.

^d^Standard deviation values (*n* = 2).

## Discussion

The total non-digestibility of GOS is questioned. An i*n vivo* study with neonatal rats fed with infant formula with added GOS showed a selective degradation of certain GOS in the small intestine while others remain intact^7^. Also, a study evaluating the *in vivo* ileal digestibility of GOS in growing rats (5 weeks old) revealed that the trisaccharide fraction was susceptible to small intestinal partial hydrolysis, having a relatively high digestibility rate (~53%)^8^. Concretely, that study showed that the glycosidic linkages (1 → 6) and (1 → 2) between galactose and glucose monomers were significantly more resistant to *in vivo* gastrointestinal digestion than the linkage (1 → 4) between galactose units in GOS with DP ≥3. In line with previous evidence, GOS having β-(1 → 6) as the predominant linkage showed a significantly higher overall resistance (23% of hydrolysis degree) to *in vitro* intestinal digestion, using a rat small intestinal extract under physiological conditions of temperature and pH, than GOS whose main linkage was β-(1 → 4) (34% of hydrolysis degree), highlighting the key role played by the glycosidic linkage involved in the oligosaccharide chain^9^. Moreover, GOS, having β-(1 → 4) as the predominant linkage, added to milk were partially digested (35%) using a similar rat intestinal extract^10^. These findings clearly pointed out a different susceptibility of GOS to mammalian digestive enzymes depending on their structural features.

In previous studies of prebiotic properties of GOS with different glycosidic linkage composition, synthesized by β-galactosidases from several probiotic bacteria, the growth rates of those probiotic bacteria were higher in GOS produced by using their own β-galactosidases^22–24^. In this context, gathering knowledge around the transglycosylation mechanism of mammalian small intestinal β-galactosidase could unravel the catalytic activity towards the hydrolysis of the different linkages present in GOS. Our results have demonstrated that pig small intestinal β-galactosidase preferably synthesizes GOS linked by β-(1 → 3) bonds, since the major di- and trisaccharide were produced by the transfer of a galactose unit to the C-3 of the non-reducing moiety of lactose and to the C-3 of glucose, respectively (Structures 1 and 2 in Fig. 2). In addition, the versatility of the pig enzymes embedded in the small intestinal BBMV was barely shown by the identification of minor trisaccharides and disaccharide formed by the transfer of galactose to glucose moiety through β-(1 → 2) or β-(1 ↔ 1) glycosidic linkages (Table 3), as well as by the scarce presence of GOS having β-(1 → 6) glycosidic linkages. Strikingly, no galactosyl-galactoses (galactobioses) could be identified. Furthermore, the mechanism of GOS synthesis was consistent for the three isolated small intestinal BBMV, revealing the lack of inter-individual variability in the studied pigs (Fig. 1B). Only quantitative differences, especially at shorter reaction times, in GOS production were observed (Table 1) and those were easily explained by the different β-galactosidase activity values determined for each of the tested BBMV.

To the best of our knowledge, our data are the first evidence on the transglycosylation activity of mammalian small intestinal glycosidases. Particularly, the gathering of knowledge on the transglycosylation mechanism of mammalian small intestinal β-galactosidase should help to design tailored fully non-digestible GOS. According to the reported data, dietary GOS tri- and disaccharides having β-(1 → 4), β-(1 → 2), β-(1 ↔ 1) or β-(1 → 6) as predominant glycosidic linkages might be less prone to hydrolysis by mammalian intestinal digestive enzymes as compared to those linked by β-(1 → 3). Likewise, regardless of the type of glycosidic linkage, galactosyl-galactoses might be more resistant to hydrolysis than galactosyl-glucoses, strengthening the prebiotic role of the former.

The mammalian small intestinal brush border membrane is situated at the luminal pole of the enterocyte where it constitutes a functional organelle promoting terminal digestion and absorption of the end products of ingested food^25^. Concretely, carbohydrates can be digested by a wide range of intestinal disaccharidases (mainly, α-glucosidases and β-galactosidases) which are involved in different protein complex structures located in the microvillus brush border membrane of the mammalian small intestine and whose activity may depend on animal age^26,27^. Therefore, the approach followed in this work could be extended to other substrates different from lactose for gaining knowledge of the mechanism of action of other intestinal glycosidases. Such information could be crucial for future studies investigating the structure-function relationship of the great variety of potentially available prebiotics, as well as for deciphering their role as modulators of the gut microbiota composition and activity.

## Methods

### Preparation of pig small intestinal brush border membrane vesicles (BBMV)

BBMV were obtained following the methodology previously proposed^28,29^. Briefly, three pig small intestines, from the duodenum to the ileum, were obtained from a local slaughterhouse (Coca, Spain). Pigs were post-weaned and 7–10 months old. Immediately after sacrifice, the samples were kept at 4 °C and transferred to the laboratory in less than 2 hours. The small intestines were rinsed with cold phosphate buffered saline solution (PBS) (pH 7.3–Oxoid; Basingstoke, UK), then slit open and scrapped with a glass slide. The mucosal scrapped was suspended (1:1, w/v) in 50 mM mannitol dissolved in PBS at 4 °C, homogenized during 10 min using a Ultra-Turrax^®^ (IKA T18 Basic), adjusted with CaCl_2_ to a final concentration of 10 mM and centrifuged at 3,000 × *g* during 30 min. The supernatant was centrifuged at 27,000 × *g* during 40 min and the resulting pellet, containing the BBMV, was re-suspended in buffer maleate (50 mM) pH 6.0 containing CaCl_2_ (2 mM) and sodium azide (0.02%). Samples were lyophilized and kept at −80 °C.

### Synthesis of galactooligosaccharides (GOS) derived from lactose using BBMV

GOS were synthesized using lactose in PBS, pH 7.3 (250 mg/mL) and the three individual lyophilized BBMV (180 mg/mL). Enzymatic reactions were carried out by duplicate for each individual BBMV samples at 37 °C in an orbital shaker at 900 rpm. Samples aliquots were withdrawn at specific time intervals (0, 2, 4 and 6 h), immediately immersed in boiling water for 5 minutes to inactivate the enzyme and centrifuged at 6,700 × *g* for 5 min at room temperature to remove BBMV. Samples were stored at −20 °C for subsequent analysis.

β-galactosidase activity is expressed in units per gram (U/g) where 1 unit is defined as the amount of enzyme hydrolysing 1 μmol of lactose per minute under the assayed conditions. β-galactosidase activities for BBMV 1, BBMV 2 and BBMV 3 were 12.08, 12.61 and 16.05 U/g, respectively.

In order to discard the presence of hydrolytic/synthesis action derived from lysosomal β-galactosidase potentially present in the three individual lyophilized BBMV, a parallel synthesis was carried out using lactose (250 mg/mL) in PBS (pH 7.3) containing *p*-chloromercuribenzoic acid (CMB) (0.1 mM), a well-known inhibitor of this type of β-galactosidase but not for those located in the BBMV^30^. Same chromatographic profiles were obtained with and without CMB, indicating that GOS synthesis was exclusively due to the action of the β-galactosidase embedded in the small intestinal BBMV (chromatograms comparison is shown in Figure S12).

### Quantification of carbohydrates in enzymatic mixtures by Gas Chromatography-Flame Ionization Detector (GC –FID)

The carbohydrates present in the mixture and purified GOS were analysed by GC-FID as trimethylsilylated oximes (TMSO) prepared following the method of Brobst and Lott^31^. Carbohydrate oximes were formed by adding 250 μL of hydroxylamine chloride in pyridine (2.5% w/v) to dried samples and heating the mixture at 70 °C for 30 min. The resulting oximes were silylated with hexamethyldisilazane (250 μL) and trifluoroacetic acid (25 μL) at 50 °C for 30 min. The reaction mixtures were centrifuged at 6,700 × *g* for 2 min at room temperature. Supernatants were injected into the GC-FID or stored at 4 °C prior to analysis.

Separation of carbohydrates was carried out in an Agilent Technologies gas chromatograph (Mod 7890A) equipped with a flame ionization detector (FID) and a fused silica capillary column DB-5HT (5%-phenyl-methylpolysiloxane; 30 m × 0.25 mm × 0.10 µm) (Agilent) following the method of Cardelle-Cobas *et al*.^32^. The oven temperature was initially set at 150 °C then programmed to 380 °C at 3 °C/min. The injector and detector temperatures were set at 280 and 385 °C, respectively. Injections were carried out in split mode (1:20) using nitrogen at 1 mL/min as the carrier gas. Data acquisition and integration were performed using the Agilent ChemStation software. Quantification was performed by the internal standard method using phenyl-β-glucoside (0.5 mg/mL) and the corresponding response factors from solutions containing glucose, galactose, lactose and raffinose (used as a standard of trisaccharides). Carbohydrates concentration was expressed as mg/100 mg of initial lactose and percentage weight of the total carbohydrates within the same polymerization degree. All analyses were carried out in duplicate and the data were expressed as means ± standard deviation (SD).

### Purification and isolation of GOS synthesized by BBMV

#### Activated charcoal treatment

In order to remove monosaccharides, GOS mixture obtained after 6 h of reaction was purified with activated charcoal (powder, Sigma-Aldrich, St. Louis, USA) following the methodology proposed by Hernandez *et al*.^33^. Briefly, GOS mixture (500 mg) and activated charcoal (3 g) were added to 100 mL of ethanol (5% v/v). The mixture was stirred for 30 min at 25 °C and then filtered through Whatman No.1 paper under vacuum. Desorption of oligosaccharides from the activated charcoal was carried out with 100 mL of ethanol (50%, v/v). The mixture was stirred for 30 min and filtered.

#### Isolation of synthesized GOS by HILIC-RID

Once monosaccharides were removed with activated charcoal, disaccharides and trisaccharides fractions were isolated by Hydrophilic Interaction Liquid Chromatography coupled to a refractive index detector (HILIC-RID) using a polyamine column (250 × 4.6 mm, 5 μm particle size) (YMC America, Allentown, USA). The sample was repeatedly injected and eluted with acetonitrile:water (70:30, v/v) at a 1 mL/min flow rate, fractions of di- and trisaccharides, corresponding to the main synthesized GOS, were collected, evaporated in a rotatory evaporator and lyophilized for subsequent NMR characterization.

### Structural characterisation of synthesized, purified and isolated GOS

#### Gas Chromatography-Mass Spectrometry (GC-MS)

TMSO carbohydrates derivatives were analysed using a Hewlett-Packard 6890 gas chromatograph coupled to a 5973 quadrupole mass detector (Agilent, Palo Alto, CA, USA). Separation was carried out in a fused silica capillary column DB-5HT (5%-phenyl-methylpolysiloxane; 30 m × 0.25 mm × 0.10 µm) (Agilent). The oven temperature was initially set at 150 °C then programmed to 300 °C at 3 °C/min and kept for 10 min. Injector temperature was 300 °C. Injections were carried out in split mode (1:20) using helium as carrier gas (0.8 mL/min). Mass spectrometer was operated in electronic impact (EI) mode at 70 eV with a scanning range comprised between 35 and 700 *m/z*. Interface and source temperature were 280 °C and 230 °C, respectively. Acquisition was obtained using a HPChem Station software (Hewlett-Packard, Palo Alto, CA, USA).

Identification of TMSO derivatives of carbohydrates was carried out by comparison of their mass spectra with those of standard compounds previously derivatised. Characteristic mass spectra and data reported in the literature were used for identification of those carbohydrates non available as commercial standards. These identifications were considered as tentative.

#### Nuclear Magnetic Resonance (NMR)

Structure elucidation of the purified fractions DP2 and DP3 was accomplished by Nuclear Magnetic Resonance spectroscopy (NMR). NMR spectra were recorded at 298 K, using D_2_O as solvent, on a Varian SYSTEM 500 NMR spectrometer (^1^H 500 MHz, ^13^C 125 MHz) equipped with a 5-mm HCN cold probe. Chemical shifts of ^1^H (δ_H_) and ^13^C (δ_C_) in parts per million were determined relative to internal standards of sodium [2, 2, 3, 3-^2^H_4_]−3-(trimethylsilyl)-propanoate in D_2_O (δ_H_ 0.00) and 1,4-dioxane (δ_C_ 67.40) in D_2_O, respectively. One-dimensional (1D) NMR experiments (^1^H, and ^13^C) were performed using standard Varian pulse sequences. Two-dimensional (2D) [^1^H, ^1^H] NMR experiments [gradient correlation spectroscopy (gCOSY), total correlation spectroscopy (TOCSY), and rotating-frame Overhauser effect spectroscopy (ROESY)] were carried out with the following parameters: delay time of 1 s, spectral width of 2694 Hz in both dimensions, 2048 complex points in t2, 4 transients (16 for ROESY) for each of 128 time increments, and linear prediction to 256. The data were zero-filled to 2048 × 2048 real points. 2D [^1^H−^13^C] NMR experiments [gradient heteronuclear single-quantum coherence (gHSQC) and gradient heteronuclear multiple-bond correlation (gHMBC)] used the same ^1^H spectral window, a ^13^C spectral window of 30166 Hz, 1 s of relaxation delay, 1024 data points, and 128 time increments, with a linear prediction to 256. The data were zero-filled to 2048 × 2048 real points. Typical numbers of transients per increment were 8 and 32, respectively.

## Supplementary information

Complete Dataset

## Data Availability

All data generated or analysed during this study are included in this published article (and its Supplementary Information files).
