# Supplementary material for: Trans-β-galactosidase activity of pig enzymes embedded in the small intestinal brush border membrane vesicles
Source: Sci Rep. 2019 Jan 30;9:960. doi: 10.1038/s41598-018-37582-8 (PMC6353940; doi:10.1038/s41598-018-37582-8)
Supplement: Supplementary file 1 — Complete Dataset [file 41598_2018_37582_MOESM1_ESM.pdf]

Supporting Information

Trans-β-galactosidase activity of pig enzymes embedded in the small intestinal brush border membrane vesicles

Lesbia Cristina Julio-González[1], Oswaldo Hernandez-Hernandez[1], F. Javier Moreno*[1], Agustín Olano[1], Maria Luisa Jimeno[2], Nieves Corzo[1]

[1] Instituto de Investigación en Ciencias de la Alimentación, CIAL (CSIC-UAM), CEI (UAM+CSIC), Nicolás Cabrera 9, 28049 Madrid, Spain

[2] Centro de Quimica Organica "Lora Tamayo" (CSIC), Juan de la Cierva 3, 28006, Madrid, Spain

* Correspondence and requests for materials should be addressed to F.J.M. (email: javier.moreno@csic.es.

Table of Contents

Figure S1. ^{1}H NMR (500 MHz, D2O) of DP2 fraction

Figure S2. ^{13}C NMR (125 MHz, D2O) of DP2 fraction

Figure S3. gCOSY and TOCSY (500 MHz, D2O) of DP2 fraction

Figure S4. Multiplicity-edited gHSQC and gHMBC (500 MHz, D2O) of DP2 fraction

Figure S5. ^{1}H NMR (500 MHz, D2O) of DP3 fraction

Figure S6. ^{13}C NMR (125 MHz, D2O) of DP3 fraction

Figure S7. gCOSY and TOCSY (500 MHz, D2O) of DP3 fraction

Figure S8. Multiplicity-edited gHSQC and gHMBC (500 MHz, D2O) of DP3 fraction

Figure S9. ROESY (500 MHz, D2O) of DP3 fraction

Figure S10. ^{1}H (500 MHz, D2O) anomeric region of DP3 fraction

Figure S11. GC-MS spectra of disaccharide TMS oximes obtained during GOS synthesis with BBMV.

Figure S12. GC-FID profiles of the GOS trisaccharide fraction synthesized by BBMV (A) in presence or (B) absence of p-chloromercuribenzoic acid (CMB)

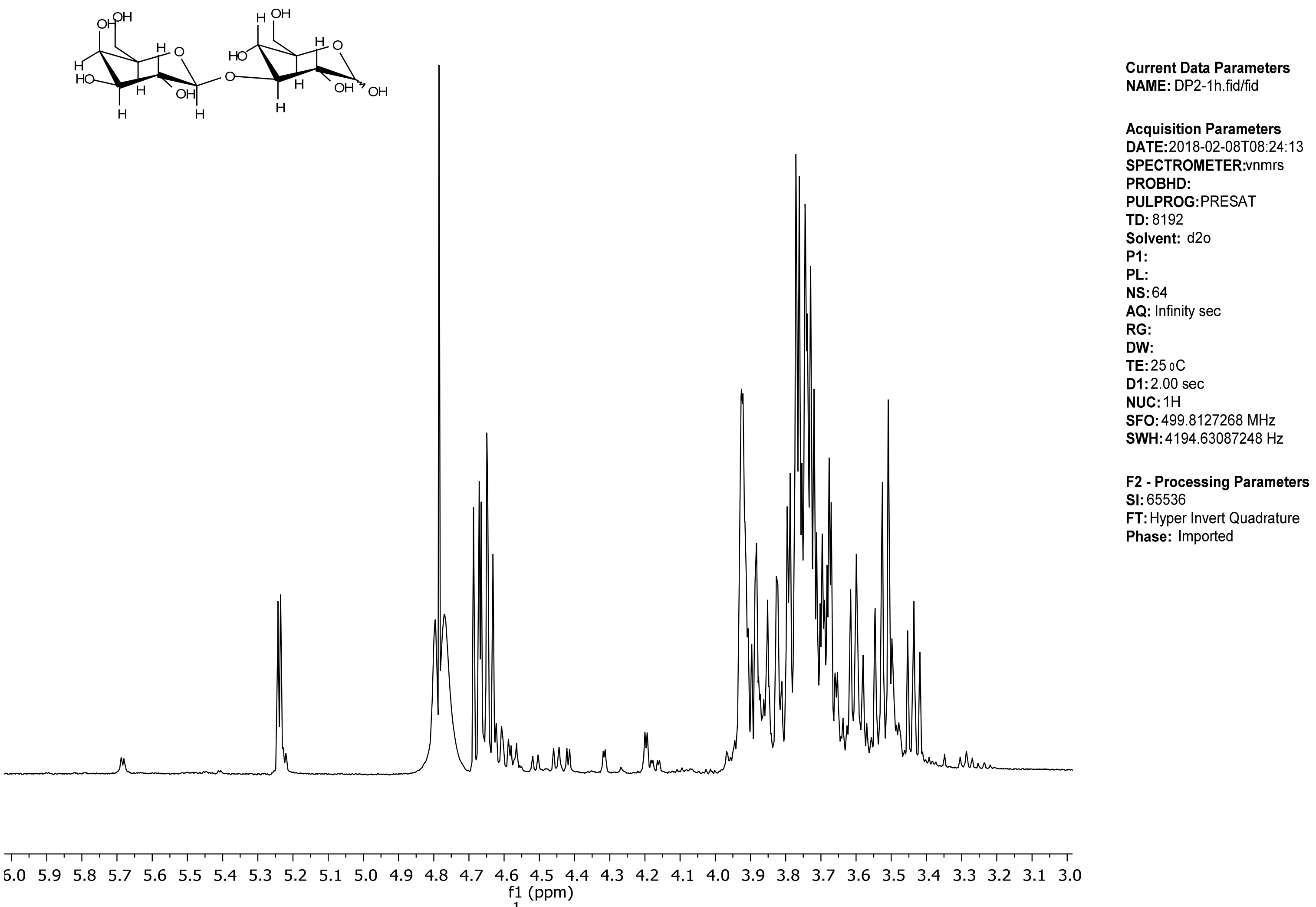

Figure S1. ^{1}H NMR (500 MHz, D2O) of **DP2** fraction

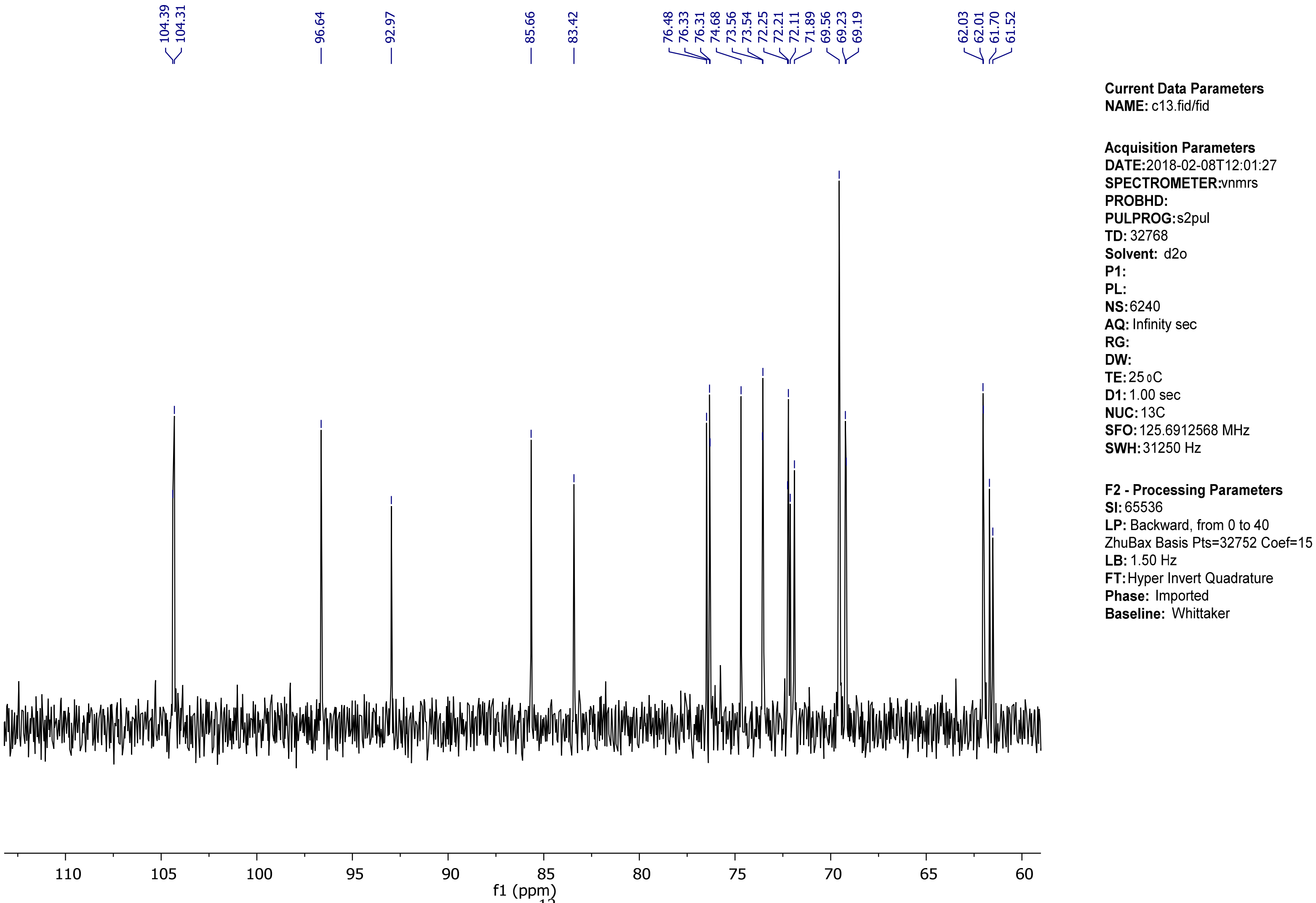

Figure S2. ^{13}C NMR (125 MHz, D2O) of **DP2** fraction

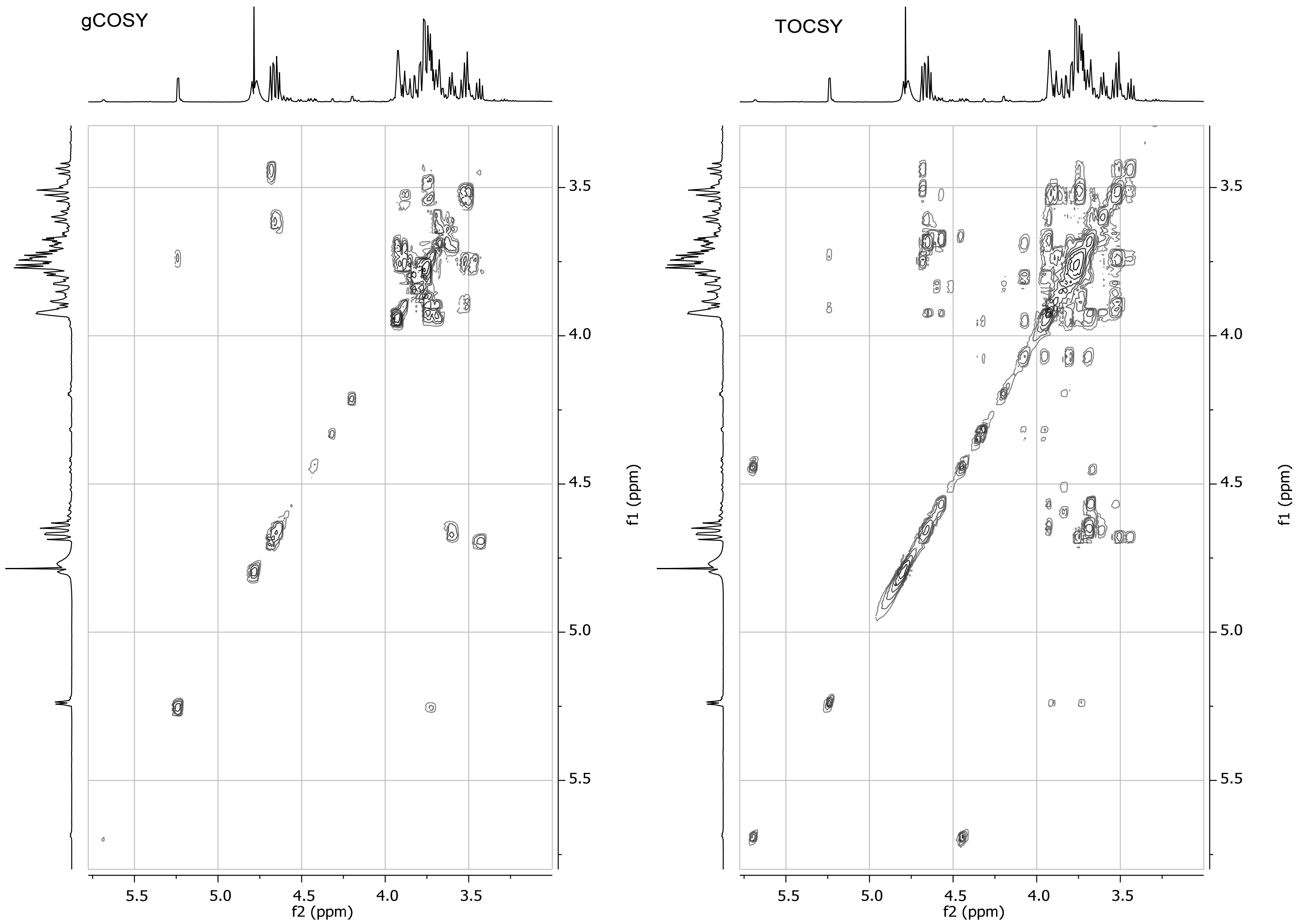

Figure S3. gCOSY and TOCSY (500 MHz, D2O) of **DP2** fraction

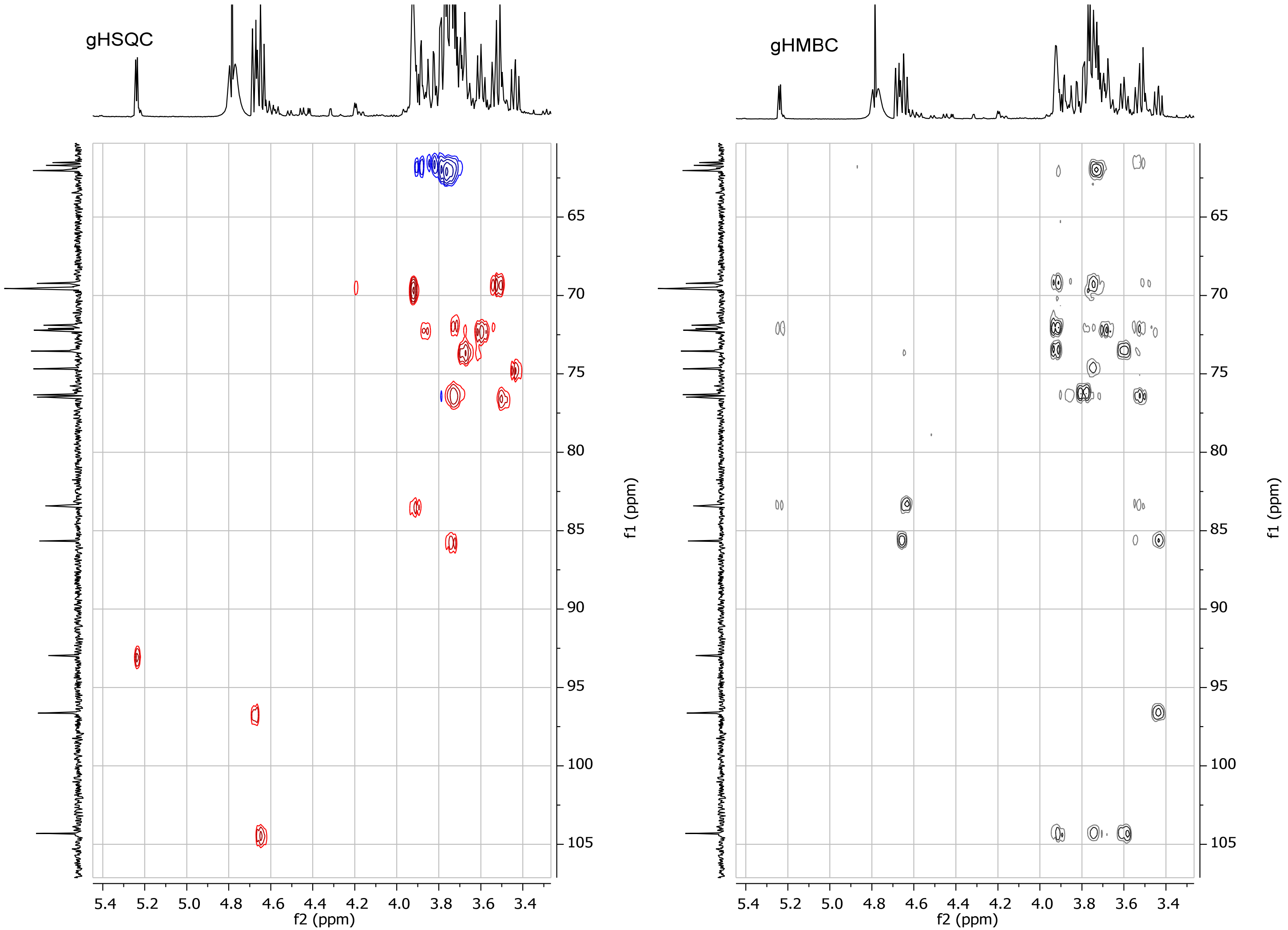

Figure S4. Multiplicity-edited gHSQC (methylene**:** blue cross peaks; methine**:** red cross peaks) and gHMBC (500 MHz, D2O) of **DP2** fraction

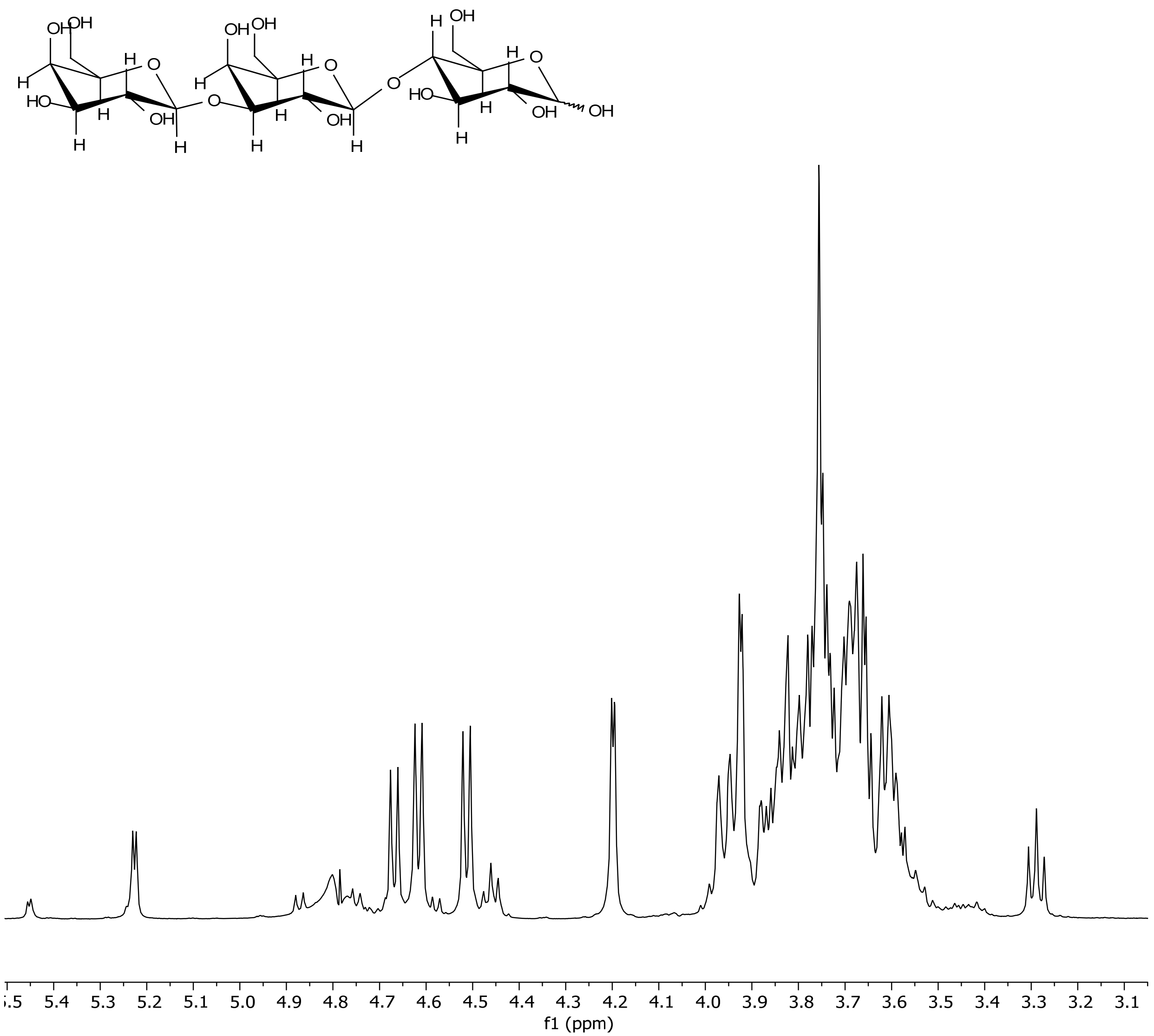

Current Data Parameters
NAME: DP3-presat-1h.fid/fid

Acquisition Parameters
DATE: 2018-01-16T16:34:59
SPECTROMETER: vnmrs
PROBHD:
PULPROG: PRESAT
TD: 8192
Solvent: d2o
P1:
PL:
NS: 32
AQ: Infinity sec
RG:
DW:
TE: 25 oC
D1: 2.00 sec
NUC: 1H
SFO: 499.8118705 MHz
SWH: 2693.96551724 Hz

F2 - Processing Parameters
SI: 65536
FT: Hyper Invert Quadrature
Phase: Imported

Figure S5. ^{1}H NMR (500 MHz, D2O) of **DP3** fraction

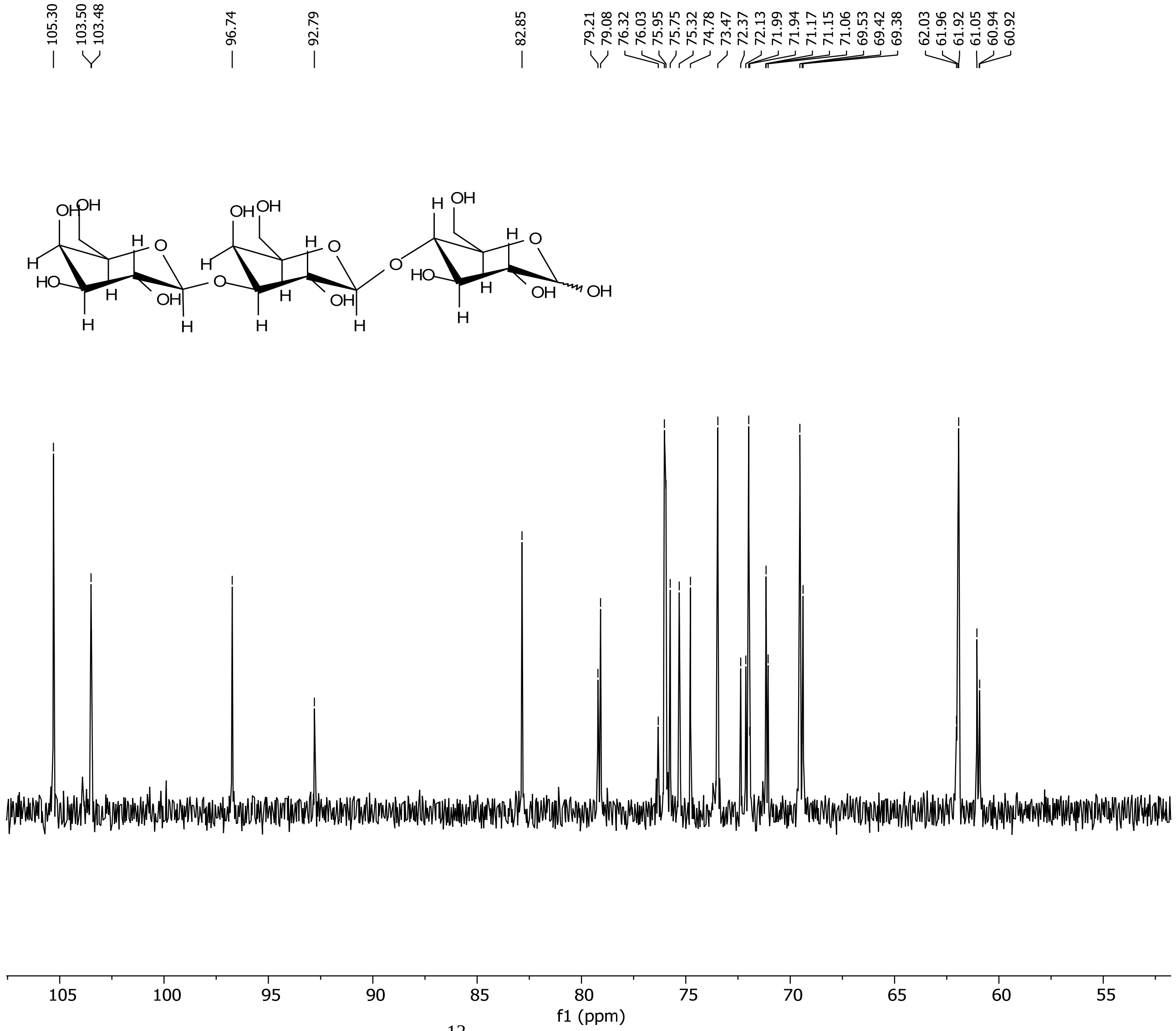

Current Data Parameters
NAME: DP3-13c-1.fid/fid

Acquisition Parameters
DATE: 2018-01-16T14:43:58
SPECTROMETER: vnmrs
PROBHD:
PULPROG: s2pul
TD: 32768
Solvent: d2o
P1:
PL:
NS: 3072
AQ: Infinity sec
RG:
DW:
TE: 25 oC
D1: 1.00 sec
NUC: 13C
SFO: 125.6912568 MHz
SWH: 31250 Hz

F2 - Processing Parameters
SI: 65536
LB: 1.00 Hz
FT: Hyper Invert Quadrature
Phase: Imported

Figure S6. ^{13}C NMR (125 MHz, D2O) of **DP3** fraction

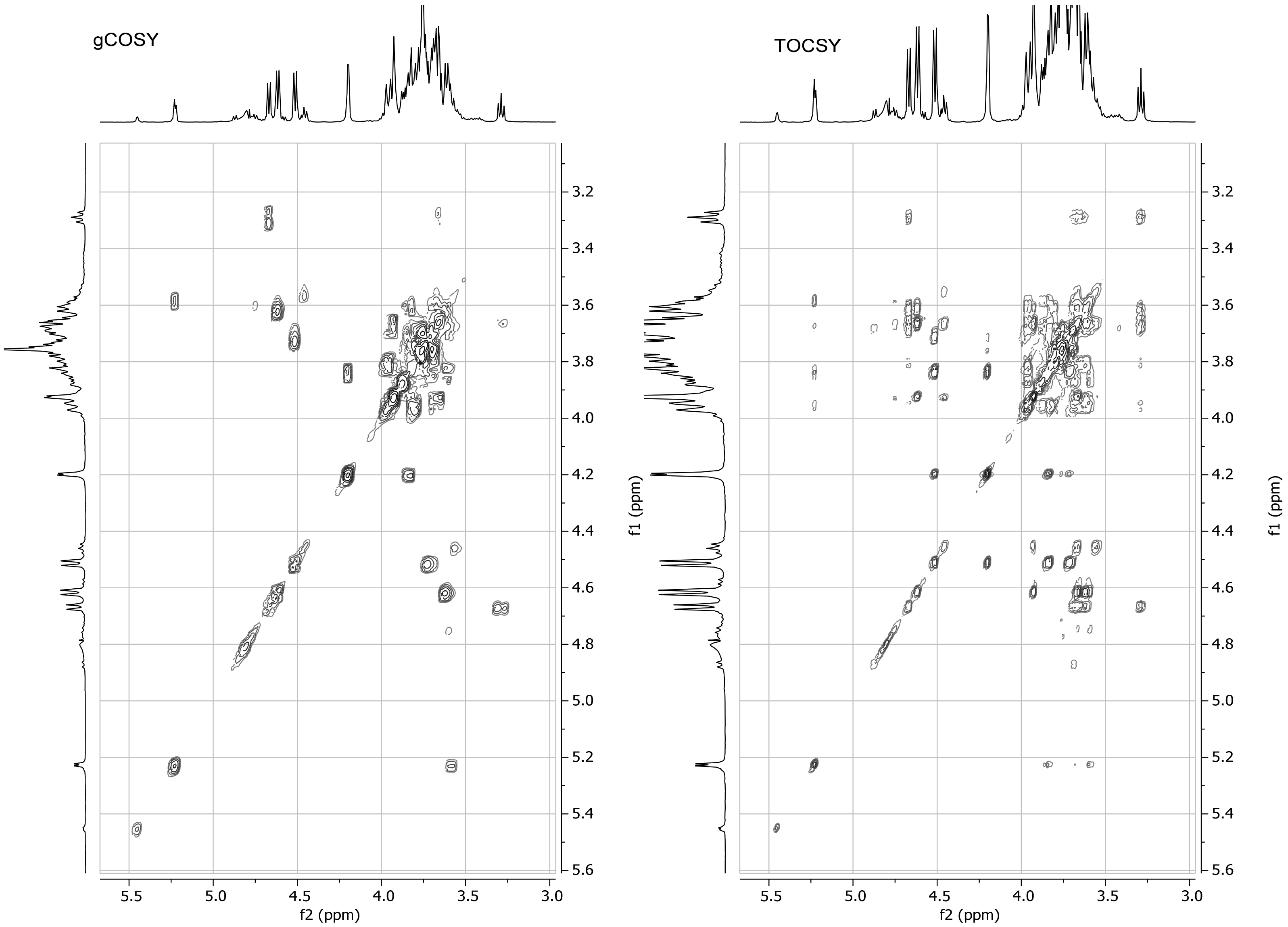

Figure S7. gCOSY and TOCSY (500 MHz, D2O) of **DP3** fraction

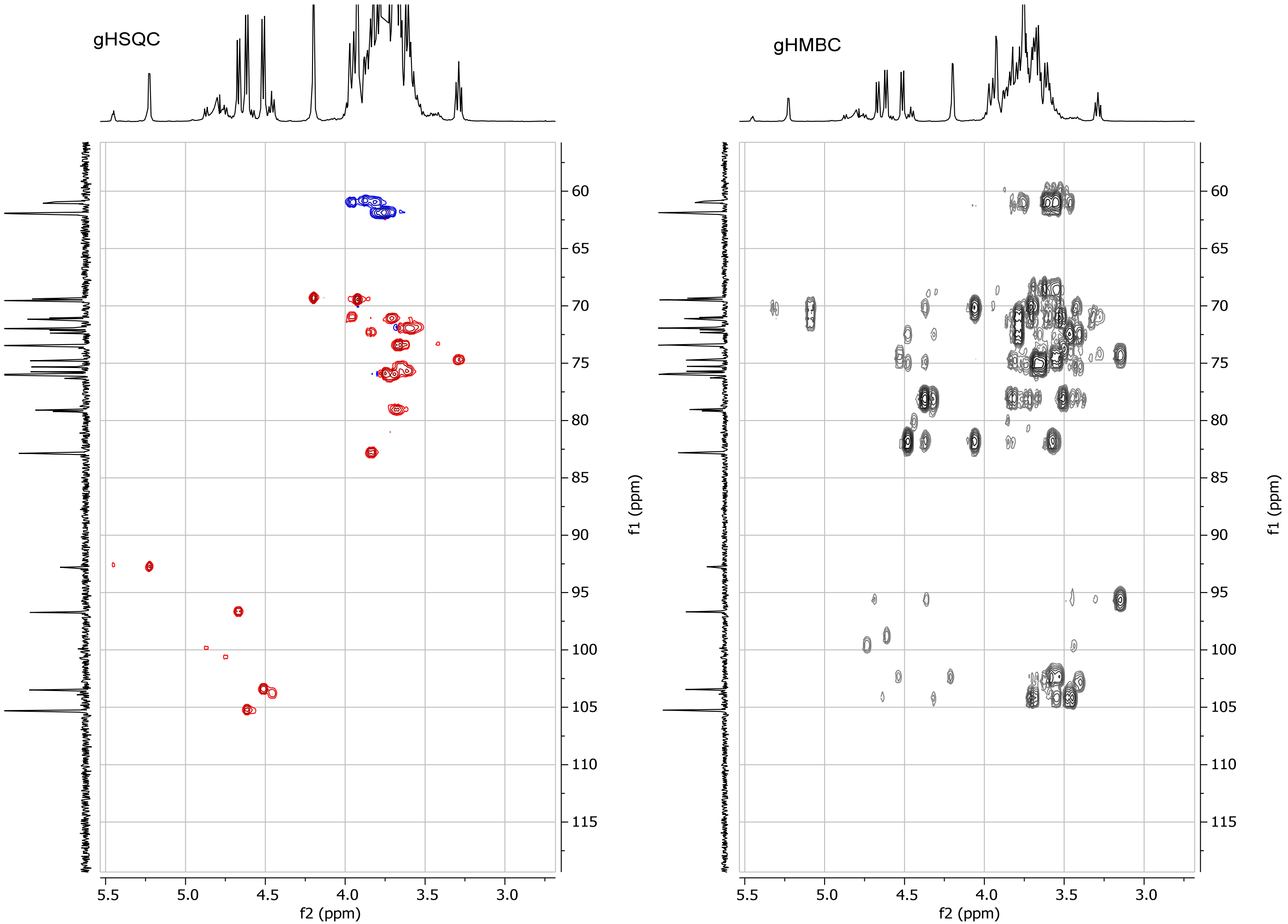

Figure S8. Multiplicity-edited gHSQC (methylene: blue cross peaks; methine: red cross peaks) and gHMBC (500 MHz, D2O) of **DP3** fraction

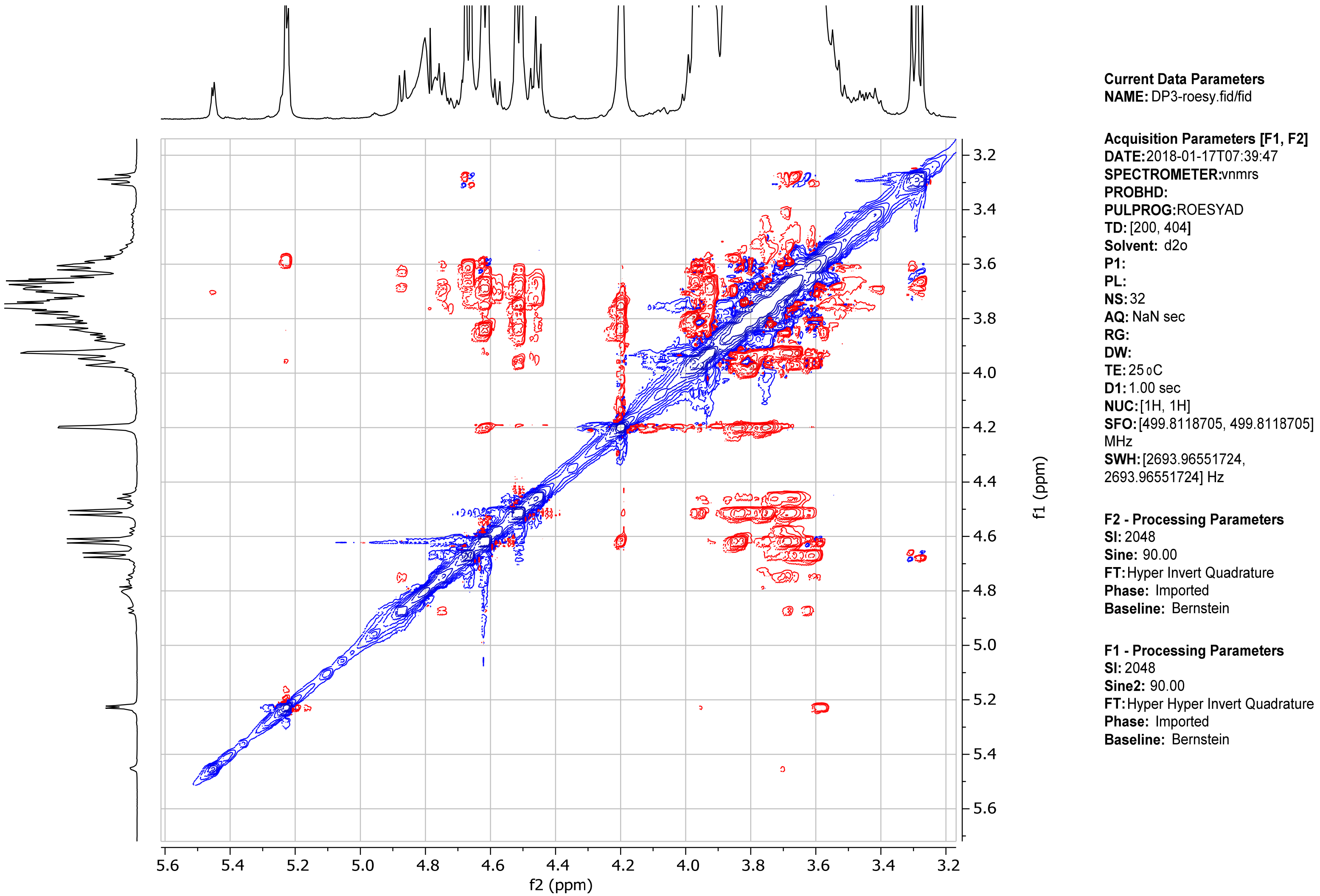

Figure S9. ROESY (500 MHz, D2O) of **DP3** fraction

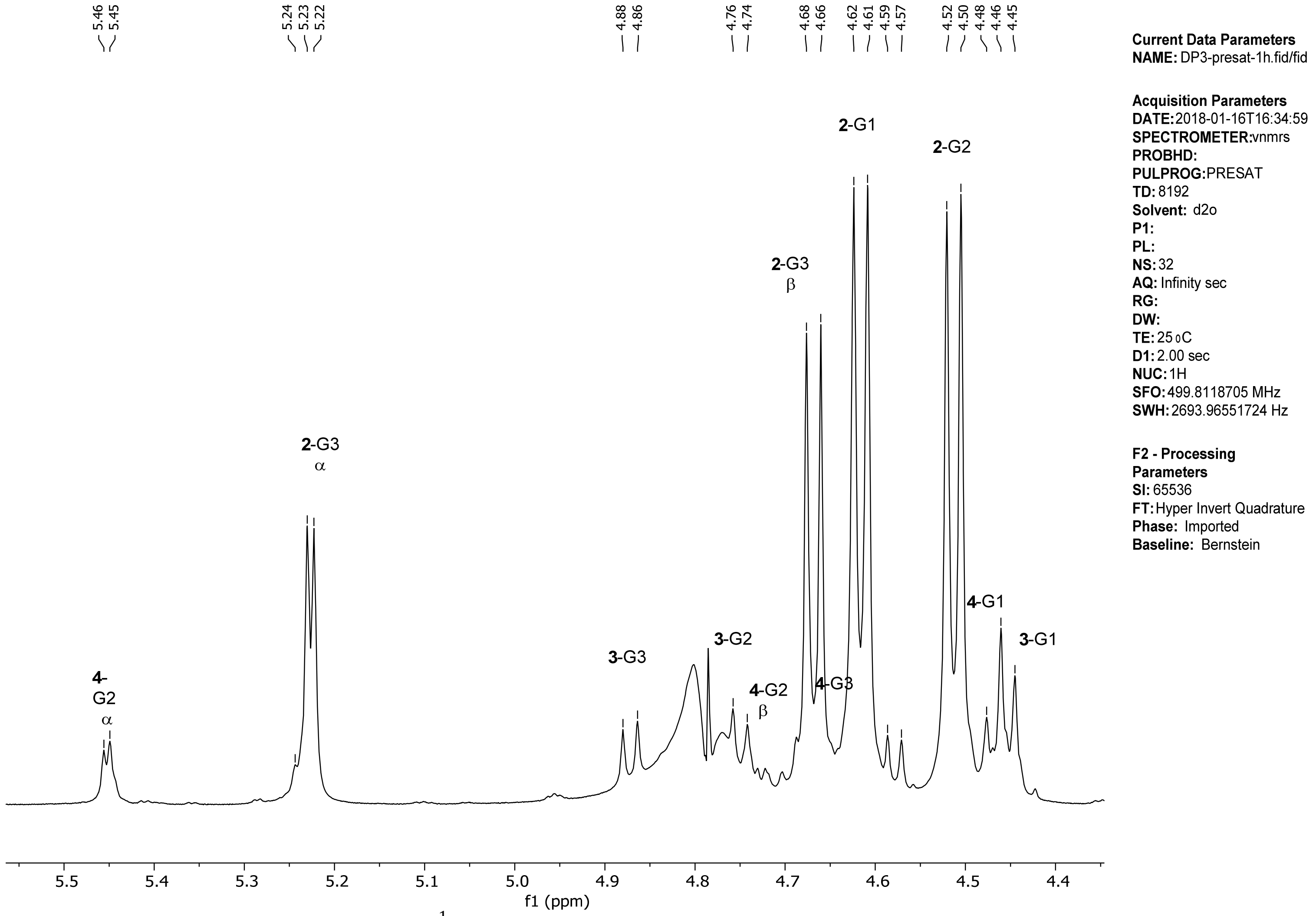

Figure S10. ^{1}H (500 MHz, D2O) anomeric region of **DP3** fraction

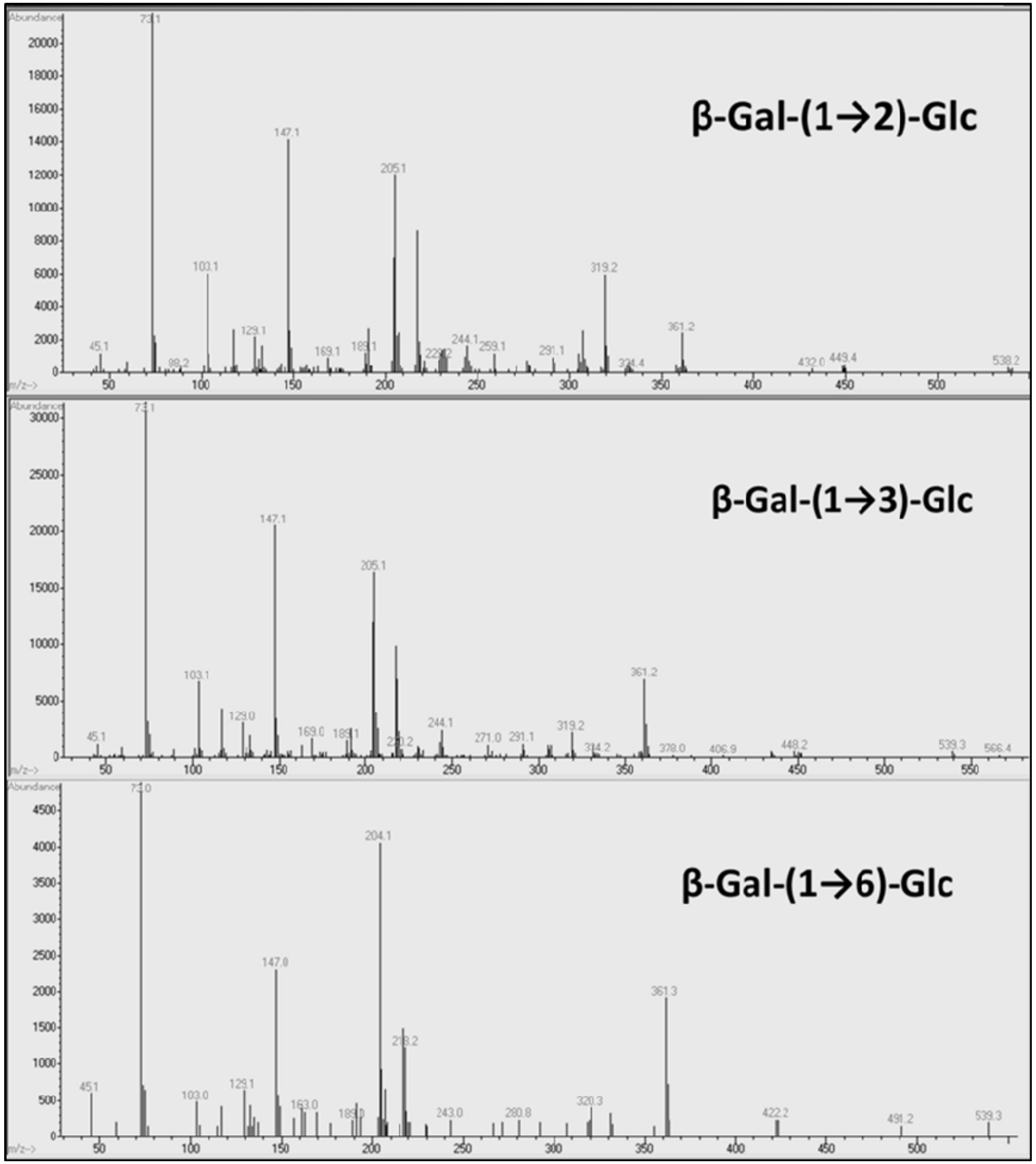

Figure S11. GC-MS spectra of disaccharide TMS oximes obtained during GOS synthesis with BBMV

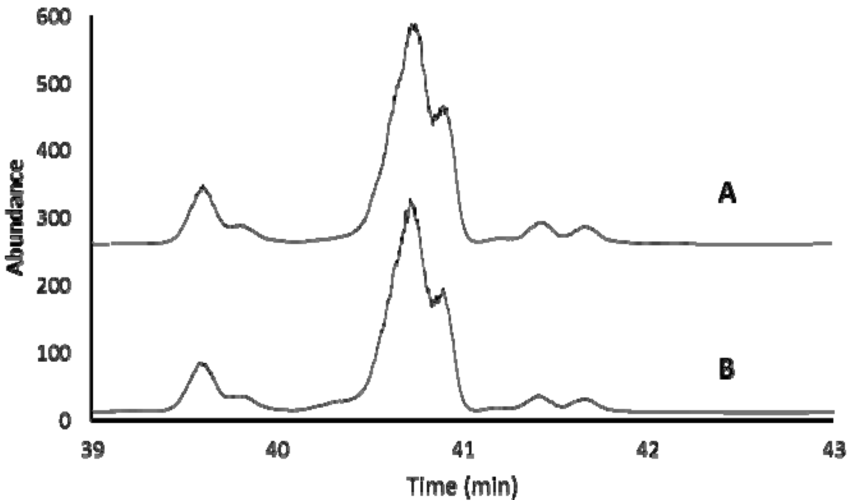

Figure S12. GC-FID profiles of the GOS trisaccharide fraction synthesized by BBMV (A) in presence or (B) absence of *p*-chloromercuribenzoic acid (CMB)
